# Analysis of complications in 97 periprosthetic Vancouver B2 fractures treated either by internal fixation or revision arthroplasty

**DOI:** 10.1007/s00402-024-05223-7

**Published:** 2024-02-24

**Authors:** H. Eckardt, D. Windischbauer, M. Morgenstern, K. Stoffel, M. Clauss

**Affiliations:** 1 Department of Orthopedics and Trauma Surgery, Basel University Hospital, Basel, Switzerland; 2 Crossklinik, Clinic for Orthopaedic Surgery and Sports Medicine, Basel, Switzerland; 3 Center for Musculoskeletal Infections, Basel University Hospital, Basel, Switzerland

**Keywords:** Periprosthetic fracture, Vancouver B2, Revision arthroplasty, Osteosynthesis, Periprosthetic fracture complication

## Abstract

**Introduction:**

The treatment of Vancouver B2 periprosthetic fractures after hip arthroplasty is still a matter of debate. Revision Arthroplasty (RA) was long thought to be the treatment of choice, however several recent papers suggested that Open Reduction and Internal Fixation (ORIF) is a viable option for selected B2 fractures. Complication rates of 14–26% have been reported following surgical treatment of B2 fractures. No significant difference between RA and ORIF in the complication rates nor in the functional outcome was observed.

**Method:**

We conducted a retrospective analysis of 97 consecutive Vancouver B2 fractures treated according to the algorithm at our institution from 2007 to 2020 and recorded complications and patient specific data.

**Result:**

From the 97 patient, 45 fractures were treated with RA while 52 fractures were treated with ORIF. Thirteen patients in the RA group had a complication that needed revision (28%) and 11 patients in the ORIF group needed revision (21%). There was no significant difference between complication rates. The reason for failure in the 13 RA patients were infection (*n* = 4), stem subsidence (*n* = 1), refracture after a new fall (*n* = 3), secondary dislocation of the greater trochanter (*n* = 1) and dislocation (*n* = 4). The reason for failure in the 11 ORIF patients that were revised were infection (*n* = 5), persistent symptomatic stem loosening (*n* = 3) and refracture (*n* = 3) after a new fall.

**Conclusion:**

ORIF can be used to revise cemented and non-cemented shafts in more than half of Vancouver B2 fractures with no difference in complication rates when compared to RA. A periprosthetic fracture around the hip has a 21–28% risk of a re-operation after revision surgery with infection and re-fracture after a new fall being the most frequent cause of re-operation.

## Introduction

Periprosthetic fractures (PPFx) after total hip arthroplasty (THA) were the second most frequent cause of revision arthroplasty (RA) in 2014 in the US with a 36% increase since 2002 and an estimated 70% increase until 2030 [1]. Overall, between 0,4% and 4% of primary arthroplasties are revised due to a periprosthetic fracture, dependant on follow-up period and completeness of the registry [2] Vancouver A fractures, which only affect the trochanteric region, can be treated conservatively if the displacement is only minimal. Vancouver B fractures affecting the meta/diaphyseal area around the stem are divided into B1 fractures with intact stem fixation, B2 fractures with stem-loosening and good bone quality, as well as B3 fractures with a loose stem and poor bone quality/multifragmentary fracture pattern. There is consent about the treatment of Vancouver B1 and B3 fractures. Vancouver B1 fractures are mostly treated with open reduction and internal fixation (ORIF) with cerclage alone or cerclage and plate fixation. The standard treatment for Vancouver B3 fractures is RA. Since the bone quality is poor and the prosthesis is loose, there is a need for a new stem with a secure diaphyseal anchoring. However, the treatment of Vancouver B2 fractures is still a matter of debate. Previously, RA was thought to be the treatment of choice for Vancouver B2 fractures, however several recent papers have challenged this dogma and suggested that ORIF is a viable option for selected B2 fractures. Complication rates of up to 14–26% after treatment of B2 fractures have been reported with no significant difference observed between RA and ORIF. In addition, functional outcome was the same when the two treatment groups were compared [2–10]. 

The controversy between the treatment option RA or ORIF for Vancouver B2 fractures is also a controversy between surgeons with a trauma background and surgeons with an arthroplasty background. In our institution, the senior surgeons managing periprosthetic fractures had predominantly trauma background. Patients with a simple periprosthetic Vancouver B2 fracture, either cemented or uncemented were treated with anatomical reduction and ORIF, whereas patients with loss of the medial stem support and those with an obviously broken cement-mantle were treated with a long non-cemented revision stem with or without additional cerclage augmentation. The aim of this paper was to compare the complications between RA and ORIF using the above-mentioned algorithm.

## Methods

### Patient cohort

From the hospital electronic patient system all patients that were treated due to a periprosthetic femur fracture between 2007 and 2020, were identified. Further inclusion criteria included availability of preoperative data regarding date of primary arthroplasty, pre-fracture mobility of the patient (not mobile; only indoor; limited outside mobile; no limitations), use of walking aids, living situation (living at home; living in a nursing home) and cognitive impairment with a diagnosis of dementia and ASA-score. The study was conducted in accordance with the Declaration of Helsinki and was approved by the local ethical committee (EKNZ 2023-00029).

Preoperative radiographs were analysed for prosthesis anchoring with or without cement. The senior authors (HE, MC) independently analysed pre and postoperative radiographs for Vancouver classification, mode of failure and operative strategy. From the electronic records, follow-up data regarding complications and reoperations were retrieved.

### Surgery

Anteroposterior and lateral radiographs of the extremity with the PPFx were analysed preoperatively for suitability for either treatment option: RA or ORIF. In most cases, an additional computer tomography was used for the decision process. Hereto it must be said that ORIF was chosen as a treatment option in fractures with a broken cement mantle around a cemented polished stem that could be adequately reduced and stabilized. These fractures provide optimal conditions for a stable stem fixation and are probably the periprosthetic fractures with the best prognosis after osteosynthesis. Periprosthetic fractures around a non-cemented stem that can be anatomically reduced and stabilized, also have a good chance of stable stem fixation. However, fractures with a broken cement mantle around a composite beam stem are often more challenging to restore using ORIF, and ORIF was only performed in patients with simple fracture patterns. Multi-fragmentary fractures, fractures with fragmented cement-mantle, fractures with severe calcar involvement and fractures around short stems were treated with RA.

The ORIF was performed in the lateral decubitus position with a lateral incision exposing the complete fracture but not opening the joint. The fracture was reduced and stabilised with cables and the construct was then stabilised with either a LISS-plate (DePuy Synthes, Oberdorf, Switzerland) (opposite side LISS turned up-side-down) or a NCB-plate (Zimmer Biomet, Zug, Switzerland) most often with a trochanter attachment plate. Essential for reduction of the fracture is the reduction of the subsided stem. Sufficient axial traction to the leg and several mallet-blows to the tip of the stem are often necessary. In low-demand patients, patients where extensive surgical procedures are contra-indicated or fractures with a simple fracture pattern proximal to the distal tip of the stem, stabilization can be achieved by 2–3 cerclages alone, needing no plate. One cerclage is fastened proximal to minor trochanter, one distal to the minor trochanter and one cerclage distal to the fracture. The cerclages can be fastened in a minimal invasive fashion.

For revision arthroplasty, the lateral decubitus position and the posterolateral exposure was used. When possible, the stem was removed through the fracture and the joint capsule was only opened as wide as necessary. A non-cemented modular revision stem (Mathys, Bettlach, Switzerland) or a non-cemented Wagner SL revision stem (Zimmer Biomet, Zug, Switzerland) were used in combination with cables for fracture reduction. A cable caudal to the most inferior fracture line was mounted before reaming the shaft.

The standard postoperative regime included mobilisation with crutches and weightbearing as tolerated after both RA and ORIF. After a few days, patients were transferred to a rehabilitation unit for 2–3 weeks or to their home/nursing home. In the outpatient clinic, patients were scheduled for routine follow-up after 6 weeks, 3 months and 12 months.

## Results

**Fig. 1:**
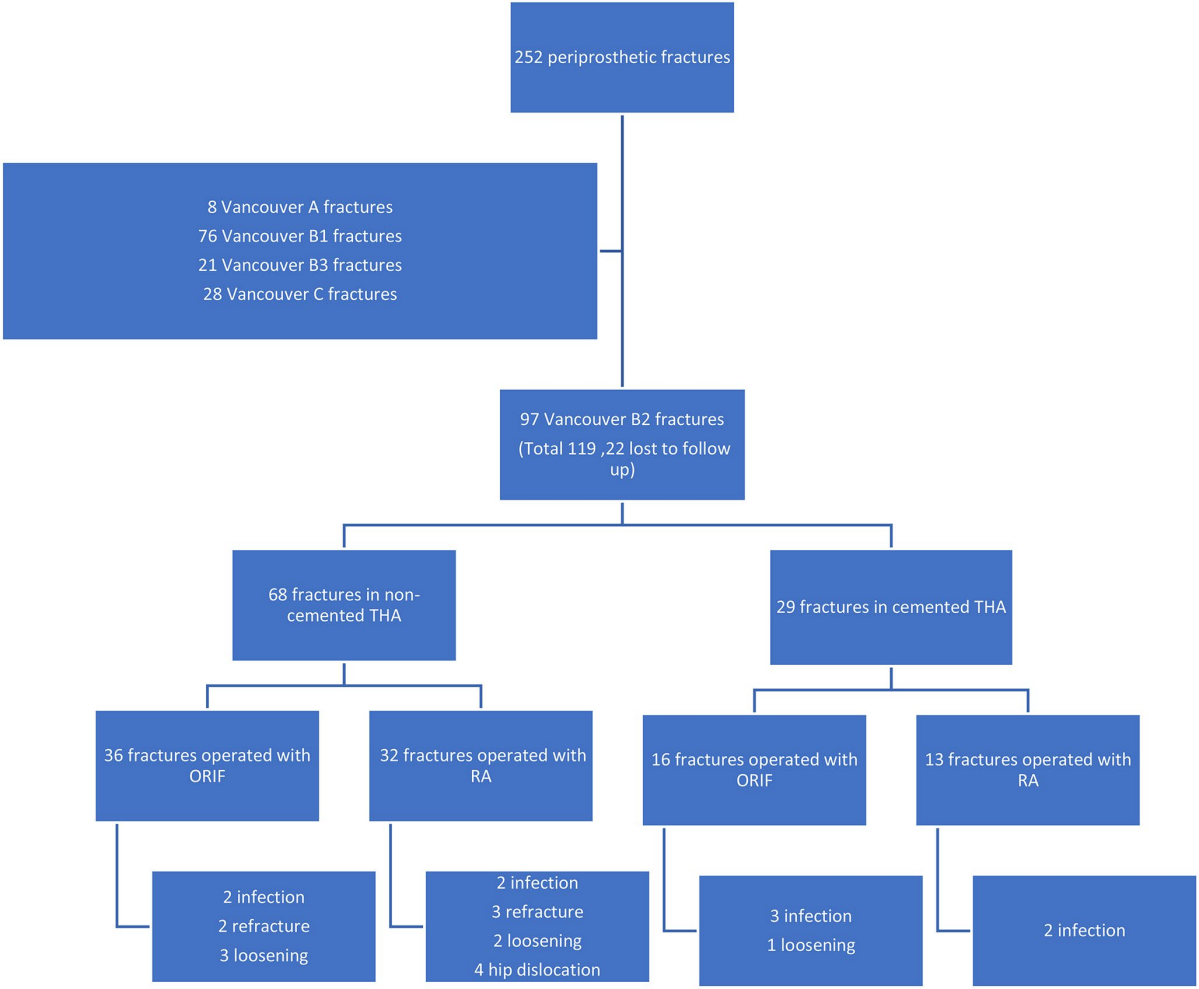
Flowchart depicting the number of periprosthetic fractures according to the Vancouver Classification, the distribution of cemented and non-cemented B2 fracture, the distribution of revision surgery and distribution of complications

252 patients with a PPFx after THA were treated between 2007 and 2020. The distribution of fractures according to the Vancouver Classification System of periprosthetic fractures, the treatment option used (RA or ORIF) and the distribution of the complications are given in Fig. 1.

Out of 119 patients with a periprosthetic Vancouver B2 fracture, 22 had one postoperative radiograph and no further follow-up. These patients were removed from the analysis. Of the remaining 97 patients, 67 patients had at least one 6-month follow-up. The mean follow-up period was 347 days in the ORIF group and 586 in the RA group.

The 97 Vancouver B2 fractures were treated with RA in 45 patients and ORIF in 52 patients (Table 1). Thirteen of the RA patients and 11 of the ORIF patients developed a complication that needed revision without significant difference between groups.

**Table 1:** Comparison of the group treated with an open reduction internal fixation (ORIF) and revision arthroplasty (RA) after periprosthetic fracture with respect to age, classification according to American Society of Anaesthesiologist, duration of operation, number of packed blood cells given during or after the operation, number of patients that was revised again, and the number of patients that developed an infection postoperative

Operation	Number of patients	Age/Years (mean) [SD]	ASA (mean)	Duration of operation/Minutes (mean)	Packed Blood Cells/Number (mean)	Revision	Infection	Patients living in nursing home	Patients mobile without walking aids
ORIF	52	80.4 [10.6]	2.6	140.6	1.04	11	5	11	14
RA	45	75.9 [11.5]	2.8	158	1.85	13	4	7	11

Number of patients living in a nursing home and whether a walking aid was needed, was recorded at the follow-up visit

**Fig. 2:**
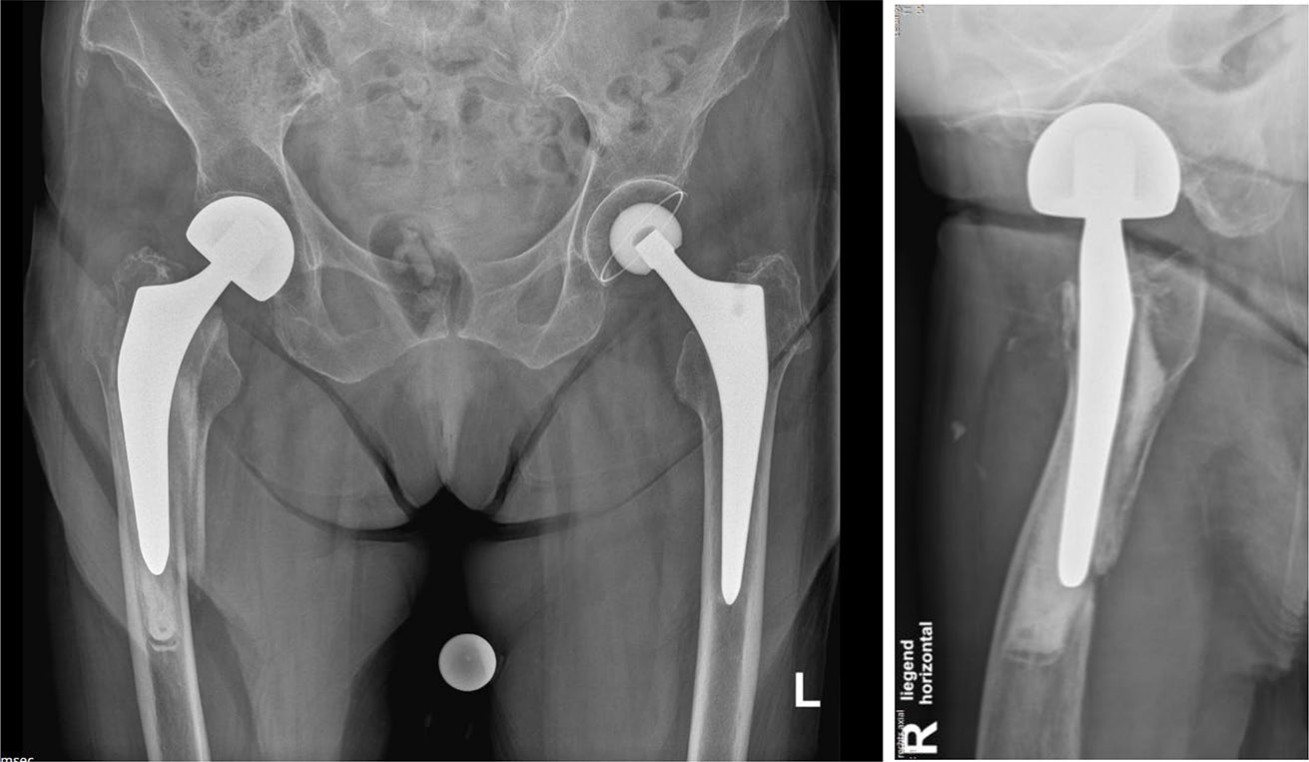
Radiograph of a right-sided Vancouver B2 fracture around a cemented polished stem. Anteroposterior view and lateral view

**Fig. 3:**
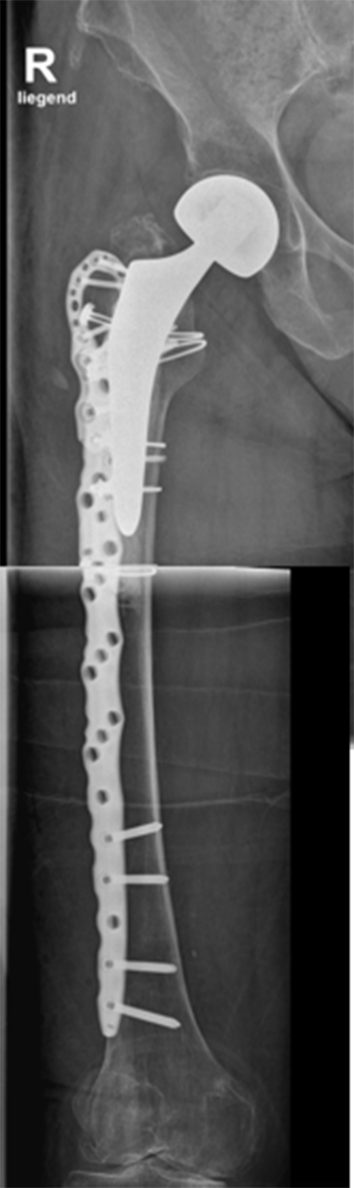
Postoperative Radiograph of the Vancouver B2 fracture depicted in Fig. 2 after open reduction and internal fixation (ORIF) with cerclage and a NCB-Plate with trochanter attachment plate

The reason for failure in the 13 RA patients were infection (*n* = 4), stem subsidence (*n* = 1), refracture (*n* = 3), secondary dislocation of the greater trochanter (*n* = 1) and dislocation (*n* = 4). The reason for failure in the 11 ORIF patients that were revised were infection (*n* = 5), persistent symptomatic stem loosening (*n* = 3) and refracture (*n* = 3) after a new fall.

The mean duration of RA surgery was 158 min (SD 52.1) while ORIF surgery was 144 min (SD 48.3; *p* = 0.13). Patients treated with RA received in the mean 1.85 packed blood cells (pbc) (SD 1.8) while patients treated with ORIF received in the mean 1.1 pbc (SD 1.53; *p* = 0.03). The mean age at the time of operation in the ORIF group was 80.4 years and 75.9 years in the RA group. The ASA score was 2.6 in the ORIF group and 2.8 in the RA group. ORIF was performed in 30 female and 22 male patients, and RA was performed in 26 female and 19 male patients. Twenty-five out of 97 patients were mobile without walking aid without a significant difference between treatment groups.

Of the 52 patients managed with ORIF, 7 patients had the fracture stabilised with cerclages only while 45 patients needed a plate and cerclages.

## Discussion

Our results highlight that more than one in five patients undergoing surgery due to a Vancouver B2 periprosthetic fracture might very well have to undergo revision surgery regardless of how the periprosthetic fracture was stabilised (RA or ORIF). The most frequent complication was infection followed by re-fracture after a new fall, dislocation and loosening. The complication rate and distribution of complications were similar to the results reported by Gausden et al. [3]. In a paper analysing reoperation rates after periprosthetic fractures, 126 Vancouver B2/3 fractures were analysed. The 2-year re-operation rate was 13% and the most frequent reasons for re-operation were infection, followed by re-fracture, dislocation and loosening. The combination of infection and periprosthetic fracture is a difficult challenge to solve with a poor prognosis [11].

**Fig. 4:**
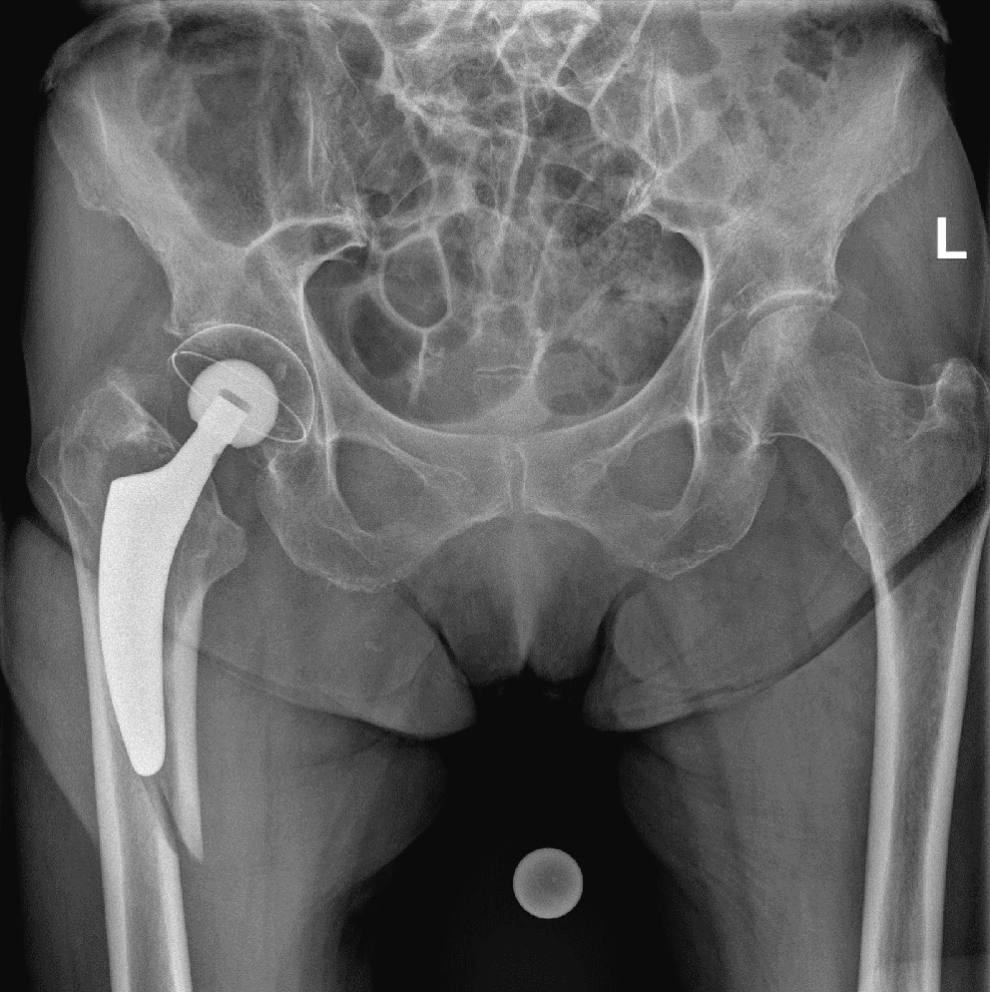
Radiograph of a right-sided Vancouver B2 fracture around a non-cemented hip arthroplasty with a short stem

**Fig. 5:**
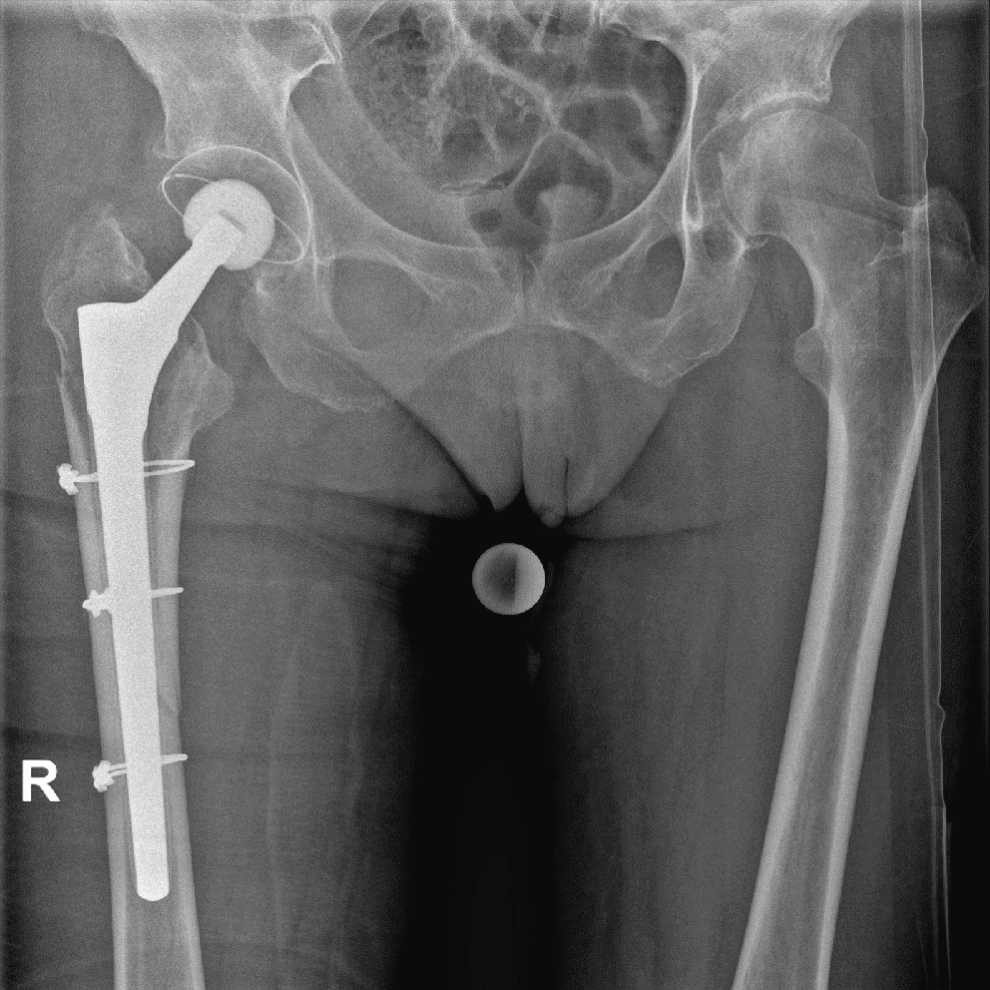
Postoperative Radiograph of the Vancouver B2 fracture depicted in Fig. 4 after revision arthroplasty with cerclage distal to the fracture and implantation of a Wagner non-cemented revision stem

Previously, RA was thought to be the only possible treatment for Vancouver B2 fractures but newer evidence suggests that ORIF is a viable treatment option in select cases [3, 6–8, 12, 13]. A recent review with pooled data from 1622 patients treated for Vancouver B2 fractures found that 21% of the patients were treated with ORIF. The remaining RA patients, received a non-cemented revision arthroplasty (57%) or a cemented arthroplasty (43%). The overall revision rate was 14% and 18% after RA and ORIF, respectively. The dislocation rate after RA was higher than after ORIF but with regard to other complications and reoperation rates, there was no significant difference between RA and ORIF [12]. Another large review with pooled data from 2509 patients included both Vancouver B2 and B3 fractures. In the subgroup analysis of patients treated for B2 fractures, the overall complication rate was 26% in patients treated with a RA and 21% in patients treated with ORIF, with no signification difference between the two. The subsidence of the stem was larger in the ORIF group than in the RA group with no difference in signs of loosening and functional outcome [6]. Both reviews conclude that ORIF is viable for the treatment of B2 fractures in select cases where fracture morphology allows a secure fixation of the stem. The authors hypothesized that in particular, low-demand patients and frail patients could profit from shorter surgery duration, lower blood loss and lower operation impact when treated with ORIF. A recent multicentre study of 184 B2 and B3 fractures around polished tapered stems operated with either RA or ORIF also showed equal revision rates in the two groups, however, the 2-year complication rate was higher after RA (25% vs. 10%) with hip dislocation being the most common complication [7]. Stoffel et al. reached the same conclusion after reviewing 14 recent studies on the treatment of Vancouver B2/B3 fractures with either RA or ORIF. The patients that had their fractures stabilised with ORIF had lower duration of operation, lower need for blood transfusion and no difference in functional outcome when compared to RA [8]. More than half the patients with a Vancouver B2 fractures in our institution were treated with ORIF. The ORIF group was 4 years older than the RA group, there was no difference in ASA score and, no difference in complication rates between the RA and ORIF group. The duration of the operation was not different but the number of blood transfusion was lower in the ORIF group. The number of patients treated with ORIF was higher in the current study than in previous papers where about one fifth of the patients are usualle treated with ORIF. The main criteria for using ORIF in our institution was a periprosthetic fracture with a maximum of 3–4 large fragments that could be reconstructed anatomically. Especially, the calcar region had to be able to be adequately stabilised with cable and with locking screws from the plate. Cemented, tapered, polished stems could often be treated with ORIF, but also non-cemented stems had a good chance of postoperative stability. We avoided ORIF in fractures around composite-beam cemented stems and also in fractures around short stems (Figs. 4, 5).

Most studies discussing this topic, state that ORIF, in case of a PPFx, could be advantageous in the old and frail patients due to the shorter operating times and lower need for pbc with ORIF [3, 8, 12, 14]. We found the need for pbc was lower in the ORIF group but duration of the operation was not different. Frail patients must be able to fully weightbear immediately. Previously, RA was preferred over ORIF to treat PPFx, allowing the patient to fully weight bear immediately after surgery. In our institution all patients fully weight bear after ORIF, only two patients suffered loosening of the stem. 25% of the patients could walk without walking aid at the follow-up control without difference between groups. The meta-analysis of 2509 patients with B2 and B3 fractures found no difference in weightbearing between RA and ORIF. This suggests that it might be safe to let all patients fully weight bear following ORIF [6].

There are inherent limitations to the present study. The retrospective nature of data collection impairs the quality of the data in comparison to a prospective study. The follow up rate was low due to the high mortality in this group of patients, where 15% die within the first year [15, 16] and the high number of patients that were unwilling to attend the follow-up visits. Since data regarding mobility, cognitive impairment and living situation are extracted from the electronic patient file, the data probably underestimated the morbidity of the patients. However, to our knowledge, the current study includes the biggest cohort of patients suffering Vancouver B2 PPFx with an analysis of failure.

## Conclusion

In our institution we treated more than half of the patients with a periprosthetic fracture with ORIF. The complication rate after periprosthetic fractures was 21–28% with no difference between patients treated with RA or ORIF. The most frequent reasons for a consecutive revision surgery after PPFx treatment was infection or refracture after a new fall.

## Data Availability

The dataset generated during the current study are available form the corresponding author on reasonable request, if relevant legislation and required data protection measures are met.
